# Adaptive servo-ventilation and mortality in patients with systolic heart failure and central sleep apnea: a single-center experience

**DOI:** 10.1007/s11325-023-02807-2

**Published:** 2023-03-15

**Authors:** Paulina Sun, Kyle Porter, Winfried Randerath, David Jarjoura, Rami Khayat

**Affiliations:** 1 The UCI Sleep Disorders Center, Division of Pulmonary and Critical Care Medicine, University of California-Irvine, 20350 SW Birch Street, Newport Beach, CA 92660 USA; 2 The Center for Biostatistics, The Ohio State University, Columbus, OH USA; 3 Institute of Pneumology at the University Cologne, Bethanien Hospital, Solingen, Germany; 4 Division of Pulmonary and Critical Care Medicine, The Sleep Heart Program, The Ohio State University, Columbus, OH USA

**Keywords:** ASV, Adaptive servo-ventilation, CSA, Central sleep apnea

## Abstract

**Background:**

Central sleep apnea (CSA) is associated with increased mortality and morbidity in patients with heart failure with reduced ejection fraction (HFrEF). Treatment of CSA with a certain type of adaptive servo-ventilation (ASV) device that targets minute ventilation (ASVmv) was found to be harmful in these patients. A newer generation of ASV devices that target peak flow (ASVpf) is presumed to have different effects on ventilation and airway patency. We analyzed our registry of patients with HFrEF-CSA to examine the effect of exposure to ASV and role of each type of ASV device on mortality.

**Methods:**

This is a retrospective cohort study in patients with HFrEF and CSA who were treated with ASV devices between 2008 and 2015 at a single institution. Mortality data were collected through the institutional data honest broker. Usage data were obtained from vendors’ and manufacturers’ servers. Median follow-up was 64 months.

**Results:**

The registry included 90 patients with HFrEF-CSA who were prescribed ASV devices. Applying a 3-h-per-night usage cutoff, we found a survival advantage at 64 months for those who used the ASV device above the cutoff (*n* = 59; survival 76%) compared to those who did not (*n* = 31; survival 49%; hazard ratio 0.44; CI 95%, 0.20 to 0.97; *P* = 0.04). The majority (*n* = 77) of patients received ASVpf devices with automatically adjusting end-expiratory pressure (EPAP) and the remainder (*n* = 13) received ASVmv devices mostly with fixed EPAP (*n* = 12). There was a trend towards a negative correlation between ASVmv with fixed EPAP and survival.

**Conclusion:**

In this population of patients with HFrEF and CSA, there was no evidence that usage of ASV devices was associated with increased mortality. However, there was evidence of differential effects of type of ASV technology on mortality.

## Introduction

Sleep disordered breathing (SDB) is the most common comorbidity in patients with heart failure (HF). The two types of SDB, central sleep apnea (CSA) and obstructive sleep apnea (OSA), have a combined estimated prevalence of 70% among patients with heart failure with a reduced left ventricular ejection fraction (HFrEF) [1–3]. In these patients, studies suggest that CSA is associated with negative outcomes, including increased mortality and readmissions [4, 5]. Initial experience suggested that continuous positive airway pressure (CPAP) would be effective for the treatment of CSA [6–9]. Subsequently, a large randomized controlled trial (RCT) found no beneficial effects of CPAP on the mortality or cardiac function in patients with HFrEF-CSA [10]. More recently, a novel ventilatory device was introduced for the treatment of CSA: adaptive servo-ventilation (ASV), which delivers end-expiratory positive airway pressure (EPAP) to maintain airway patency along with inspiratory pressure support (IPAP) and a variable backup respiratory rate to maintain regular ventilation and abort impending central apnea events [11]. Initial studies comparing ASV to CPAP in the treatment of CSA found ASV to be superior in controlling CSA [12–16]. Other studies reported improved cardiac function in patients with HFrEF-CSA treated with ASV [13, 17–21].

In 2015, SERVE-HF, a large RCT, evaluated the effect of ASV in patients with HFrEF-CSA on a composite of endpoints, including time to major cardiovascular events and mortality, and found no significant effect of ASV therapy on these specified endpoints. An exploratory analysis of cardiovascular mortality events revealed a relationship between treatment and mortality [22]. Following publication of this trial, ASV, in all its forms, has not been prescribed for HFrEF-CSA [23]. This leaves this group of patients with no widely accepted treatment modality.

SERVE-HF was an industry-sponsored RCT evaluating a single type of ASV device that targets minute ventilation (ASVmv) and does not possess the airway patency sensing capabilities and automatically adjusting end-expiratory pressure (auto-EPAP) features that more advanced devices made by the same manufacturer and others have in their ASV devices. Additionally, other types of ASV devices that deliver peak flow-targeted ASV (ASVpf) have been widely used in the treatment of CSA with positive reports on some surrogate outcomes [14, 24]. A separate RCT by the manufacturer of this ASVpf device was concurrently underway and most recently reported no harm from this ASVpf device on the same population of patients with HFrEF-CSA. Speculations about the mechanism of harm associated with this device used in the SERVE-HF trial included that the algorithm favors ventilation over airway stabilization, which may result in increased intrathoracic pressure and, thus, a pro-arrhythmogenic metabolic alkalosis [25, 26]. Attention was directed to the difference of treatment delivery across various ASV technologies.

The varying effects on safety of these devices suggest that the mechanisms of action and clinical effects of these devices are different in this population of patients with HFrEF. Unfortunately, while several small studies suggested benefits, there are minimal studies examining the effect of the device algorithm on critical physiological parameters, such as control of breathing, chemosensitivity, blood gases, and autonomic functions. Few studies have compared different ASV devices [27]. An important recent study underscored the significant differences between different ASV device algorithms on the ventilatory status in a single-night study [28].

We have previously examined a large cohort of patients with HFrEF-CSA in our center and found that device therapy, including ASV, was associated with lower, not greater, mortality in these patients [29]. Our patients were mostly treated with ASVpf devices. Therefore, we sought to further explore the question of whether or not different types of ASV were associated with different effects on mortality in our patients with HFrEF-CSA.

## Methods

### Study design and setting

Using the Ohio State University (OSU) Sleep Heart Program database, along with device manufacturer and vendor databases, we identified all patients with HF who were diagnosed with CSA and prescribed an ASV device between 2008 and 2015. Initial diagnostic and subsequent titration polysomnography were performed in the clinical OSU sleep laboratory. Event definition and scoring were executed according to the American Academy of Sleep Medicine 2007 scoring manual [30]. We planned an analysis for all our patients with HFrEF-CSA to understand the relationship between type of ASV device and hours of use and mortality. By restricting analysis to only those patients who received an ASV device, this analysis avoids the potential bias that results from the differences that may exist between patients who had the opportunity to pursue treatment after diagnosis and those who did not. Therefore, we planned to compare mortality between those who used the prescribed device and those who did not use or under-used the device. Once the cohort was established, one data coordinator obtained baseline characteristics and cardiac function status from the electronic medical records.

Treatment adherence and efficacy were verified using device manufacturers’ compliance monitoring software and vendor-provided data. The treatment status, device type, and adherence data were added to the database by another research coordinator blinded to the vital status of patients since this information was available outside the electronic medical records.

### Outcomes

Once established, the cohort was submitted to the institutional honest broker (“OSU Information Warehouse”), which interfaces with the electronic medical record and state and nationwide vital statistics on all patients in the OSU Sleep Heart Program registry. The honest broker provided the mortality data.

A close-out date was predetermined to be March 1, 2018, for the entire database. This close-out date was used to establish censored follow-up time for all patients who remained alive during the study period according to the databases.

The study protocol was approved by the Ohio State University Institutional Review Board (2007H0043 and 2007H0055). This study complies with the Declaration of Helsinki.

### Statistical analyses

We planned a primary hypothesis test which compared all-cause mortality of patients with HFrEF and concomitant CSA who were confirmed to be using ASV devices against patients with HFrEF-CSA who were classified as untreated due to lack of adherence to their prescribed ASV device. A Cox proportional hazards model was used to adjust for any differences between the treated and untreated groups on variables that were reported as being associated with mortality in HF patients, including age, sex, body mass index (BMI), apnea–hypopnea index (AHI), left ventricular ejection fraction (LVEF), and presence of comorbid conditions including diabetes, atrial fibrillation, hypertension, coronary artery disease (CAD), chronic kidney disease (CKD), diabetes, and presence of an implantable cardioverter defibrillator (ICD) [31, 32]. All analyses were performed using SAS 9.4 (SAS Inc., Cary, NC, 2013).

## Results

### Patient characteristics

From 2008 to 2015, there were 105 patients with HF who had CSA and were prescribed ASV. Of those, 90 patients had HFrEF and were the focus of the survival analysis. The baseline characteristics of all patients included in the primary analysis group are listed in Table 1.

**Table 1:** Baseline characteristics for all patients included in the primary analysis, mean (SD) or *n* (%)

Variable	All patients (*n* = 105)	LVEF ≤ 45 (*n* = 90)	*n* missing (LVEF ≤ 45)
Age — years	62.8 (± 13.2)	62.2 (± 13.6)	0
Sex, male — no. (%)	98 (93%)	85 (94%)	0
BMI — kg/m^2^	30.8 (± 7.1)	29.8 (± 6.5)	0
AHI — no. of events/hour	50.9 (± 25.0)	49.0 (± 24.6)	0
CAI — no. of events/hour	35.3 (± 31)	23 (± 24)	0
LVEF — %	32.0 (± 13.5)	28.3 (± 10.2)	0
Therapy type			0
Fixed — no. (%)	74 (71%)	66 (73%)	
Auto — no. (%)	31 (30%)	24 (27%)	
Creatinine — mg/dL	1.62 (± 1.64)	1.58 (± 1.61)	1
Atrial fibrillation — no. (%)	47 (45%)	37 (41%)	0
Hypertension — no. (%)	57 (54%)	49 (54%)	0
CAD — no. (%)	55 (52%)	47 (52%)	0
CKD — no. (%)	28 (27%)	21 (23%)	0
Diabetes — no. (%)	38 (36%)	31 (34%)	0
ICD — no. (%)	70 (67%)	64 (71%)	0

*BMI*, body mass index; *AHI*, apnea–hypopnea index; *CAI*, central apnea index; *LVEF*, left ventricular ejection fraction; *CAD*, coronary artery disease; *CKD*, chronic kidney disease; *ICD*, implantable cardioverter defibrillator

### Treatment parameters

Device settings and efficacy data are summarized in Table 2. We examined different cutoffs of usage in terms of effect on mortality and found the biggest effect was associated with a 3-h-per-night cutoff. This 3-h threshold was similarly used in SERVE-HF [22]. Therefore, we selected this cutoff for the multivariable analysis of survival. Using this threshold, there were 59 patients who could be classified as “users.” Patients who were non-adherent, “under-users,” were defined as ASV device use less than 3 h per night (*n* = 31). Of those 31 patients who under-used the device, 21 patients recorded usage less than 0.9 h per night and were further deemed “non-users” of ASV for the purpose of the sensitivity analysis addressing the relationship between device exposure and mortality.

**Table 2:** ASV treatment parameters

Measure	*N*	Median (range)	Mean (SD)
Hours of use per night used — no	90	4.4 (0–10.8)	3.9 (± 3.3)
Average EPAP — cm H_2_O	59	7.3 (4.1–14)	7.8 (± 2.6)
Average PS — cm H_2_O	70	3.5 (0.5–12)	4.2 (± 2.7)
Residual AHI — no. of events/hour	75	6.8 (0.1–37.6)	8.4 (± 7.5)
Average minimum ventilation — L/min	57	8.0 (3.3–17.9)	8.1 (± 2.5)
Average breath rate — breaths/min	68	16.2 (9.5–20)	19.1 (± 22.6)

*EPAP*, expiratory positive airway pressure; *PS*, pressure support; *AHI*, apnea–hypopnea index

Of the 59 patients who were deemed as users, the majority were prescribed ASVpf devices (*n* = 49), and the remaining 10 patients received ASVmv devices. Of the 49 patients who received ASVpf devices, only 7 had a fixed EPAP algorithm. All the patients who received ASVmv devices had a fixed EPAP unit.

### Relationship between treatment with ASV and survival in patients with HFrEF-CSA

**Fig. 1:**
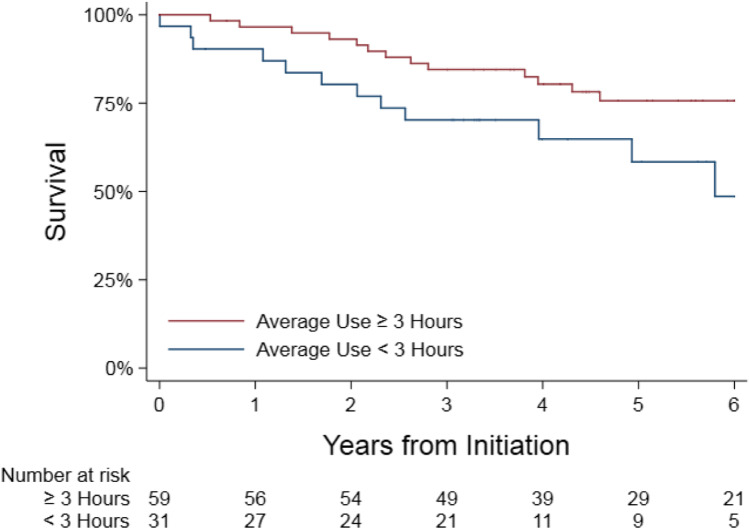
Kaplan–Meier post-discharge survival plot of patients with HFrEF-CSA by treatment status in the primary analysis including all patients. HFrEF-CSA, heart failure with reduced ejection fraction and concomitant central sleep apnea

Differences in the baseline characteristics between the two main usage groups are listed in Table 3. The unadjusted Kaplan–Meier mortality estimates are listed in Table 4 and depicted in Fig. 1. A Cox proportional hazards model was used to calculate the mortality hazard ratio for patients with HFrEF-CSA who were classified as users (*n* = 59) compared to those who did not use or under-used the treatment device (*n* = 31). The model adjusted for age, sex, BMI, AHI, LVEF, creatinine, atrial fibrillation, hypertension, CAD, CKD, diabetes, and ICD, which are all the characteristics listed in Table 1. Follow-up time started at the date of initiation of therapy based on device download data. The median follow-up time for this analysis was 64 months. The adjusted hazard ratio for mortality in the treated group (ASV users) compared to the untreated group (under-users and non-users) was 0.44 (CI 95%, 0.20 to 0.97; *P* = 0.04).

**Table 3:** Baseline patient characteristics by usage group, mean (SD) or *n* (%)

Variable	Average use < 3 h per night (*n* = 31)	Average use ≥ 3 h per night (*n* = 59)
Age — no	59.1 (± 16.0)	63.8 (± 11.9)
Sex, male — no. (%)	28 (90%)	57 (97%)
BMI — kg/m^2^	27.1 (± 5.3)	31.3 (± 6.7)
AHI — no. of events/hour	48.5 (± 22.2)	49.2 (± 25.9)
LVEF — %	26.5 (± 10.3)	29.2 (± 10.1)
Creatinine — mg/dL	1.35 (± 0.58)	1.71 (± 1.94)
Atrial fibrillation — no. (%)	9 (29%)	28 (48%)
Hypertension — no. (%)	16 (52%)	33 (56%)
CAD — no. (%)	19 (61%)	28 (48%)
CKD — no. (%)	7 (23%)	14 (24%)
Diabetes — no. (%)	12 (39%)	19 (329%)
ICD — no. (%)	20 (65%)	44 (75%)
Type of EPAP		
Auto — no. (%)	9 (29%)	42 (71%)
Fixed — no. (%)	1 (32%)	17 (29%)
Non-user — no. (%)	21 (68%)	0 (0%)
Ventilation algorithm		
ASVmv — no. (%)	0 (0%)	10 (17%)
ASVpf — no. (%)	10 (32%)	49 (83%)
Non-user — no. (%)	21 (68%)	0 (0%)

*BMI*, body mass index; *AHI*, apnea–hypopnea index; *LVEF*, left ventricular ejection fraction; *CAD*, coronary artery disease; *CKD*, chronic kidney disease; *ICD*, implantable cardioverter defibrillator; *EPAP*, expiratory positive airway pressure; *ASVmv*, minute ventilation-targeted adaptive servo-ventilation; *ASVpf*, peak flow-targeted adaptive servo-ventilation

**Table 4:** Estimated unadjusted mortality from Kaplan–Meier estimator (95% CI)

Year	Average use < 3 h per night (*n* = 31)	Average use ≥ 3 h per night (*n* = 59)
1	90%	97%
2	80%	93%
3	70%	85%
4	65%	80%
5	58%	76%
6	49%	76%

### Exploration of the effect of type of ASV device

To further explore the effect of relevant clinical variables on mortality, we calculated the univariate hazard ratio associated with each clinical variable in Table 5. Exploring the effect of auto-EPAP vs. fixed EPAP, a univariate mortality hazard ratio 3.07 (CI 95%, 1.22 to 7.74; *P* = 0.02) was found supporting the protective effect of auto-EPAP. There was no statistically significant increased impact on mortality when ASVmv devices were compared to ASVpf (HR 1.92; CI 95%, 0.77 to 4.84; *P* = 0.16). However, all 10 ASVmv devices had the fixed EPAP feature such that the two variables (ASVmv with or without fixed EPAP) could not be separated.

**Table 5:** Univariable Cox proportional hazards models for mortality

Variable	Hazard ratio	95% confidence interval	*P*-value
Average use ≥ 3 h	0.44	(0.20, 0.97)	0.04
Baseline age	1.03	(0.99, 1.06)	0.07
Sex, male	Inestimable*		
Baseline BMI	0.95	(0.89, 1.02)	0.14
Baseline AHI	1.01	(0.99, 1.03)	0.22
Baseline LVEF	0.99	(0.95, 1.03)	0.23
Baseline creatinine	1.19	(1.06, 1.35)	0.01
Atrial fibrillation	1.01	(0.46, 2.23)	0.98
Hypertension	0.76	(0.35, 1.66)	0.47
CAD	1.16	(0.53, 2.56)	0.71
CKD	2.10	(0.93, 4.76)	0.07
Diabetes	1.36	(0.61, 3.03)	0.46
ICD	2.25	(0.77, 6.57)	0.14
Type of EPAP			
Auto	Reference	Reference	
Fixed	3.07	(1.22, 7.74)	0.02
Non-user	2.39	(0.89, 6.43)	0.09
Ventilation algorithm			
ASVpf	Reference	Reference	
ASVmv	1.92	(0.77, 4.84)	0.16
Non-user	2.77	(0.98, 7.77)	0.05

^*^Only 5 females, all survived

*BMI*, body mass index; *AHI*, apnea–hypopnea index; *LVEF*, left ventricular ejection fraction; *CAD*, coronary artery disease; *CKD*, chronic kidney disease; *ICD*, implantable cardioverter defibrillator; *EPAP*, expiratory positive airway pressure; *ASVpf*, peak flow-targeted adaptive servo-ventilation; *ASVmv*, minute ventilation-targeted adaptive servo-ventilation

## Discussion

In this study, we evaluated the outcomes of all patients with HFrEF-CSA who were prescribed ASV in a single center. At the close-out date of this study, a median follow-up time of 64 months was available. There was a positive correlation between hours of exposure to ASV treatment and survival supporting the benefit of treatment of CSA in these patients. The majority of patients were prescribed ASVpf devices with auto-EPAP. A minority of patients were prescribed ASVmv with fixed EPAP. The use of fixed EPAP was associated with negative impact on mortality in univariate analysis. The sample size did not allow a multivariable exploration of the interaction between adherence, type of EPAP, and device algorithm (ASV pf vs. ASVmv). The first important finding is that the study did not show a relation between using an ASV device and increased mortality as would have been expected based on the SERVE-HF findings. The second important finding was that the protective effect, in addition to usage hours, was associated with the type of ASV device used. ASVpf (which was predominantly an auto-EPAP device) was associated with protective effect, while ASV devices with fixed EPAP (all of which were ASVmv devices) were associated with worse outcome. These findings help explain the discrepancy between the results of SERVE-HF and other studies that found no harm associated with ASV (especially ASVpf) in patients with HFrEF-CSA [14, 16, 33]. This suggests a significant difference in the physiologic effects of various ventilatory support devices that have been used interchangeably thus far in patients who are exposed to the harmful effects of excessive ventilation [25, 34]. The discrepant ventilatory effects of these devices were recently highlighted in a study that evaluated 4 ASV devices and found that they behaved differently and delivered different degrees of ventilation and EPAP in face of similar complex events [28]. The findings of this study are also consistent with reports from ADVENT-HF, a large RCT using an ASVpf as opposed to the ASVmv used in SERVE-HF, which also demonstrates these devices to be safe in patients with HFrEF-CSA.

Patients with HFrEF-CSA may manifest central apnea events with underlying obstructive pathophysiology and central apnea events without upper airway collapse, all in a changing distribution with varying severity over the course of a single night of sleep [35–37]. Therefore, successful elimination of CSA events and the associated complex breathing disorder would require variable levels of ventilation support and EPAP delivery throughout the sleep period. The premise of the ASV effect is predicated upon its proprietary sensing capabilities that distinguish between central and obstructive respiratory events and its complex multi-targeted treatment algorithms that deliver variable ventilation during central events while maintaining airway patency [11]. Therefore, both the sensing and therapeutic algorithm of ASV and its behavior across the sleep period and throughout the treatment duration of this complex shifting disorder are of paramount importance to these devices’ safety and efficacy. ASV devices have undergone several modifications since the first generation was introduced. Notable among these modifications is the addition of auto-EPAP, which improves the device’s ability to maintain the patency of the airway [27] and potentially minimizes airway-related respiratory control instability [35]. In addition, these modifications included changes to the therapy algorithms [11]. The first-generation device used in SERVE-HF utilized a fixed EPAP and a predetermined set minimal pressure support, which can potentially increase the intrathoracic pressure and ventilation in the patients with HFrEF. Such shortcomings in the algorithm of this particular device were cited as a possible explanation for the increased mortality in SERVE-HF as detailed elsewhere [11, 24].

### Limitations

In this study, we found that adherence to the prescribed ASV device was protective. Of course, the observational design likely introduces bias with the effect of adherent behavior potentially driving this observation. However, an important conclusion here is that if exposure to ASV of all its types is harmful, then this observed protective effect of adherence on mortality might have been neutral or even negative. Importantly, the sample size did not allow a comprehensive multivariable comparison between the different device algorithms and types. Exploring the clinical consequences of different types of ASV devices on patients with HFrEF will require a larger sample size with a multicenter international registry to include patients on different manufacturers’ devices, different settings and algorithms, and be able to account for the numerous covariables related to the underlying HF and SDB.

This study cannot be used to support any re-introduction of ASV for the treatment of CSA in patients with HFrEF. However, the discrepancy between the observation of benefit from treatment and lack of correlation between hours of device exposure and harm does question the conclusion that all forms of advanced positive pressure devices are harmful in this population. This study along with others that support an absence of harmful effects of ASVpf with auto-EPAP devices underscores the risks of bringing advanced ventilation devices into practice with insufficient understanding of their proprietary algorithms, its specific effects in special populations such as patients with HFrEF patients, and the possible gaps in device approval processes. While several small studies have supported the benefits of some types of ASV in this population and provided some justification for a large industry-sponsored study, there were minimal evaluations of the effect of these devices on intrathoracic pressures, cardiac conductive properties, hemodynamics, blood gases, and breathing control centers to allow accurate interpretation of the findings of SERVE-HF and other trials. It could be argued that more regulatory scrutiny of advanced ventilatory assist devices prior to approval or requirements of more publicly funded research into the mechanism of action of these devices may have averted the current void in management of our patients with HFrEF-CSA.

## Data Availability

The datasets generated during and/or analyzed during the current study are available from the corresponding author on reasonable request.
